# Approach to the Patient: Challenging Cases of Pediatric Thyrotoxicosis

**DOI:** 10.1210/clinem/dgae592

**Published:** 2024-08-27

**Authors:** Christiaan F Mooij, Nitash Zwaveling-Soonawala, Jacquelien J Hillebrand, A S Paul van Trotsenburg

**Affiliations:** Department of Pediatric Endocrinology, Emma Children's Hospital, Amsterdam University Medical Centers, University of Amsterdam, 1105 AZ Amsterdam, The Netherlands; Department of Pediatric Endocrinology, Emma Children's Hospital, Amsterdam University Medical Centers, University of Amsterdam, 1105 AZ Amsterdam, The Netherlands; Endocrine Laboratory, Department of Laboratory Medicine, Amsterdam University Medical Centers, University of Amsterdam, 1105 AZ Amsterdam, The Netherlands; Department of Pediatric Endocrinology, Emma Children's Hospital, Amsterdam University Medical Centers, University of Amsterdam, 1105 AZ Amsterdam, The Netherlands

**Keywords:** thyrotoxicosis, hyperthyroidism, Graves’ disease, antithyroid drugs, pediatric, childhood

## Abstract

Graves’ disease (GD) is the leading cause of hyperthyroidism in children. However, compared to adults, GD in children is a rare condition. In a recent guideline issued by the European Thyroid Association, the diagnostic evaluation and treatment of pediatric GD is described extensively. In this article, we go beyond the guideline and describe the potential challenges of establishing the right etiology of thyrotoxicosis in children, illustrated by cases of thyroid hormone resistance, autonomous functioning thyroid nodules, and subacute thyroiditis with a thyrotoxic phase. In addition, we report therapeutic challenges in pediatric GD such as recurrent immunological flare-ups under antithyroid drug (ATD) treatment, innovative ways to improve ATD compliance and the role of definitive treatment in persistent complaints of malaise under ATD treatment.

## Hyperthyroidism and Graves’ Disease in Pediatric Patients

In children, the primary cause of hyperthyroidism is Graves’ disease (GD). GD is an autoimmune condition that results from the presence of TSH-receptor antibodies (TRAb; also known as thyrotropin binding inhibiting immunoglobulins, abbreviated as TBII) and leads to an overactive thyroid gland (Graves’ hyperthyroidism) (1). In GD, TRAb act as an agonist of the TSH receptor, causing excessive thyroid hormone secretion and disrupting pituitary control of the thyroid (2). In adults GD is quite prevalent; in children it is a rare disease with an incidence of 4.58/100 000 per year, and the incidence is even lower under the age of 15 years (1 to 2.91/100 000 per year) (3). This is also reflected in the fact that (only) approximately 5% of all GD patients have their onset during childhood (4). Signs and symptoms of hyperthyroidism in pediatric GD are similar to those in adults, but they may also include accelerated growth and bone maturation and a decline in academic performance (5). Unfortunately, there is often diagnostic delay due to suspected behavioral, gastrointestinal, respiratory, or cardiac conditions. In 2022 the European Thyroid Association (ETA) published a guideline on the management of pediatric GD (3). Although treatment options, like antithyroid drugs (ATD), radioactive iodine (RAI), and thyroid surgery, are similar to those in adults, the advantages and disadvantages differ in the pediatric population. A prolonged course of ATD treatment for 3 to 5 years or more aiming at immunological and biochemical remission is stated to be the first-line treatment option, with a preference for dose titration compared to block and replace treatment strategy. If definitive treatment is indicated, either total thyroidectomy or RAI aiming at complete thyroid ablation is recommended. Local expertise plays an important role in the choice between thyroidectomy or RAI as definitive treatment. Current management of pediatric GD is extensively described in the 2022 ETA guideline (3).

While GD is the leading cause of primary pediatric hyperthyroidism, the differential diagnosis includes several nonautoimmune causes of thyroid hormone excess such as infectious causes (eg, subacute thyroiditis, acute bacterial thyroiditis), thyroid hormone-producing nodules (eg, autonomous functioning nodules or nodule(s) as part of McCune–Albright syndrome), familial genetic nonautoimmune hyperthyroidism (due to activating *TSHR* mutations), iodine-induced hyperthyroidism, TSH-secreting pituitary adenoma, thyroid hormone resistance, and thyrotoxicosis due to excessive intake of thyroid hormone (3). Especially in cases where the diagnosis of GD is not confirmed by the presence of TRAb, and in cases where TSH levels are not suppressed, other causes of thyrotoxicosis should be considered. In addition, laboratory assay interferences should always be considered when thyroid function tests do not match the clinical picture.

In this article we discuss challenging cases in the diagnosis and management of pediatric thyrotoxicosis that go further than the scope of the clinical ETA guideline.

### Challenging Cases of Pediatric Thyrotoxicosis—Establishing the Right Diagnosis

#### Case 1

A 15-year-old boy was referred to a general pediatrician because of abdominal pain. In the diagnostic workup, thyroid function tests were included, revealing a high plasma free T4 (FT4) concentration [96.6 pmol/L; Roche (Cobas), reference interval (RI) 12-22] and a normal TSH [1.16 mU/L; Roche (Cobas), RI 0.5-5]. Additional diagnostics for thyroid hormone excess revealed normal total T4, thyroxine-binding globulin and total T3 levels. Serum TRAb and anti-thyroperoxidase (TPO) antibodies were negative and remained negative during follow-up. Thyroid ultrasound showed a small but otherwise normal thyroid gland, and thyroid scintigraphy showed homogenous uptake. TRAb-negative GD was suspected, and the boy was treated with methimazole. The methimazole dose was increased because of persistent high FT4 levels (51.2-64.6–38.2-42.2 pmol/L); TSH levels, however, remained normal (1.40-1.21–0.95-1.58 mU/L). Because the boy developed progressive tiredness and headaches, methimazole treatment was stopped after 6 months, and after consultation with a pediatric endocrinologist at our national and European center of expertise for rare thyroid disorders, the following differential diagnosis was considered: generalized thyroid hormone resistance, TSH-producing pituitary adenoma, or a false biochemical picture of thyroid hormone excess due to assay interference. Genetic testing ruled out a mutation in the *THRB* gene, and a normal pituitary magnetic resonance imaging scan and low plasma alpha subunits ruled out a TSH-producing pituitary adenoma. Eventually the boy was referred to our clinic for further workup, and initial biochemical analysis revealed completely normal thyroid hormone levels [FT4 14.7 pmol/L (Delfia, PerkinElmer, RI 10-23); TSH 1.8 mU/L (Cobas, Roche, RI 0.5-5]. In hindsight, the previous abnormal thyroid function tests were attributed to assay interference.

This case illustrates that an incorrect diagnosis of GD may have a great impact on the clinical course of the patient. The fact that GD is the most common cause of pediatric thyrotoxicosis probably played a role in the initial clinical management in this case. The major argument to rule out GD as a differential diagnosis in this boy with “increased” FT4 levels was the absence of suppressed TSH levels. Both analytical assay interference and several true disorders (eg, thyroid hormone resistance, monocarboxylate transporter 8 deficiency, TSH-secreting pituitary adenoma) may cause the biochemical profile of raised FT4 levels with nonsuppressed TSH (6). The absence of TRAb and true signs and symptoms of hyperthyroidism should have been other triggers to reconsider the diagnosis of GD in this case. Hormones, like FT4 and TSH, are measured using immunoassays, which may rather frequently suffer from assay interferences (0.4-4%) caused by a wide variety of causes including abnormal thyroid hormone binding proteins; antibodies to, for example, iodothyronines, TSH, or streptavidin; other heterophilic antibodies; or (excessive) biotin supplementation (6-8). Consultation of a clinical chemist-endocrinologist to rule out assay interference and to perform biochemical analysis of thyroid hormone levels using a different assay should have been the first step to rule out true hyperthyroidism and can prevent unnecessary diagnostic tests and treatment (6). If testing on another assay platform does not solve the problem, several additional analytical methods to rule out assay interference may be considered, like measuring free thyroid hormone concentrations following equilibrium dialysis and detection of possible interfering compounds (6).

#### Case 2

An 8-year-old girl was referred to the pediatric endocrinologist by her general practitioner after biochemical evaluation because of weight loss revealed a high FT4 (48 pmol/L; RI 12-22) with a slightly increased TSH (5.6 mU/L; RI 0.6-4.8). At presentation, her weight had been stable for 3 months, and there were no signs or symptoms related to thyrotoxicosis. Biochemical reevaluation confirmed high thyroid hormone concentrations [FT4 49.8 pmol/L, free T3 13 pmol/L (RI 3.0-6.2)] with a slightly increased TSH (5.4 mU/L). TRAb and anti-TPO antibodies were negative. Additional thyroid scintigraphy showed homogenous uptake. Initially, GD was suspected despite the nonsuppressed TSH and absence of TRAb. As the girl had no signs or symptoms of thyrotoxicosis, treatment with methimazole was not yet started and the diagnostic workup of high FT4 levels with normal TSH levels was expanded, confirming slightly increased TSH levels using a different assay and excluding macro-TSH. Next, genetic testing for thyroid hormone resistance was performed. A heterozygous pathogenic variant in the *THRB* gene was identified [c.949G > A; p.(Ala317Thr)], confirming the diagnosis of generalized thyroid hormone resistance. Since there were no clinical symptoms of either thyroid hormone receptor beta resistance or thyroid hormone receptor alpha overstimulation (explained later), treatment was not started.

Resistance to thyroid hormone action due to mutations in the thyroid hormone receptor beta-isoform (TRβ) is biochemically characterized by elevated FT4 levels without TSH suppression. The nonsuppressed TSH is explained by the expression of TRβ in the pituitary. Symptomatology is characterized by a hypothyroid state of tissues expressing TRβ and a thyrotoxic state of tissues predominantly expressing TR alpha. For example, TR alpha is strongly expressed in cardiac tissue, which may lead to tachycardia and atrial fibrillation in case of TRβ resistance. Most patients with *THRB* mutations show no signs and symptoms of thyrotoxicosis, although the clinical phenotype may differ among individuals and between different tissues in the same person due to different expressions of the mutant protein in various tissues (9). Treatment is only indicated in symptomatic cases and should be individually tailored depending on the clinical symptoms. A recent study conducted in 61 individuals with genetically confirmed *THRB* mutations showed increased risks for all-cause mortality [hazard ratio (HR) 2.84], atrial fibrillation (HR 10.56), heart failure (HR 6.35), and major adverse cardiovascular events (HR 3.49) compared to controls (10). The authors suggest that these increased risks may be driven by lifelong cardiac exposure to thyroid hormone excess and suggest regular monitoring of cardiac health and modification of other cardiovascular risk factors if present (10). Future clinical trials evaluating the effectiveness of treatment with the thyroid hormone analogue TRIAC aiming at controlling adverse cardiac outcomes and studies in larger cohorts of patients with thyroid hormone resistance are needed to better understand the potential cardiac consequences of untreated patients (10, 11).

Case 1 and case 2 both illustrate that increased FT4 levels in the presence of normal or increased TSH levels do not fit the diagnosis of GD. The specific biochemical profile and individual signs and symptoms at presentation should guide the treating physician in the decision to perform additional diagnostic tests to identify the cause of increased FT4 levels. Table 1 shows the various causes of thyroid hormone excess based on its typical biochemical profile.

**Table 1.** The differential diagnosis of pediatric thyroid hormone excess based on the biochemical profile

Elevated FT4/FT3 and suppressed TSH	Elevated FT4/FT3 and normal/increased TSH
Graves’ disease***^a^***	TSH-secreting pituitary adenoma
Hyperthyroid phase of Hashimoto's disease	Thyroid hormone resistance—inactivating mutation in *THRB^b^*
Subacute thyroiditis (de Quervain thyroiditis)—with elevated ESR/CRP***^c^***	Assay interference (biotin, anti-streptavidin, heterophilic antibodies)*^d^*
Autonomous functioning nodule—somatic activating mutation in *TSHR*, *GNAS,* or *EXH1;* sporadic toxic adenoma; hyperfunctioning thyroid carcinoma***^e^***	
McCune-Albright syndrome—mutation in *GNAS* causing (multi)nodular toxic goiter	
Familial nonautoimmune hyperthyroidism—germline-activating *TSHR* mutation	
Iodine induced hyperthyroidism	
Factitious thyrotoxicosis due to excessive intake of thyroid hormones (biochemical workup shows low serum Tg)	
Assay interference (biotin, anti-streptavidin)	

Abbreviations: CRP, C-reactive protein; ESR, erythrocyte sedimentation rate; FT3, free T3; FT4, free T4; Tg, thyroglobulin.

^*a*^Illustrated by cases 5, 6, and 7.

^*b*^Illustrated by case 2.

^*c*^Illustrated by case 4.

^*d*^Illustrated by case 1.

^*e*^Illustrated by case 3.

#### Case 3

**Figure 1.**
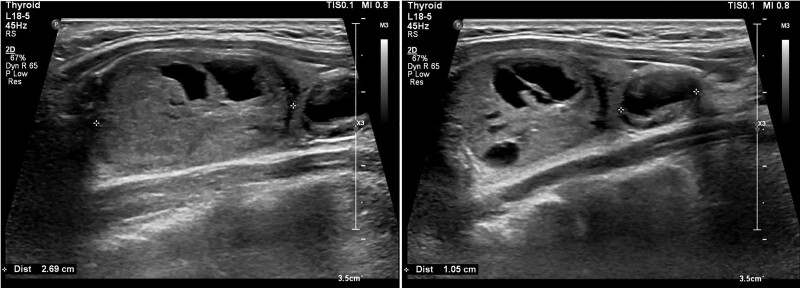
Thyroid ultrasound showing 2 nodules in the right lobe of a 13-year-old girl with autonomous functioning thyroid nodules due to somatic activating *TSHR* mutation.

A 13-year-old girl was evaluated by her general practitioner because of tiredness and swelling in the neck. At physical examination tachycardia (heart rate 115 beats/minute) was observed. Palpation of the neck revealed palpable nodules that seemed to be localized within the thyroid. Biochemical analysis showed mild thyrotoxicosis (FT4 24.6 pmol/L; RI 12-22, TSH <0.01 mU/L; RI 0.5-5) and low inflammatory markers (C-reactive protein and erythrocyte sedimentation rate). The girl was referred to our outpatient clinic for further diagnostics of thyrotoxicosis. Additional biochemical evaluation showed persistent mild hyperthyroidism (TSH 0.01 mU/L, FT4 22.7 pmol/L, T3 2.9 nmol/L; RI 1.3-2.7) and was negative for the presence of anti-TPO antibodies, anti-thyroglobulin antibodies, and TRAb. Thyroid ultrasound showed 2 nodules in the right lobe, with a diameter of 1.9 × 1.6 × 2.7 cm and 1.1 × 0.7 × 1.1 cm, respectively (Fig. 1), with a benign appearance. Scintigraphy showed focal uptake at the location of the 2 nodules in the right thyroid lobe and no uptake in the rest of the thyroid, fitting with the diagnosis of autonomous functioning thyroid nodules. Two months later, the patient underwent a hemithyroidectomy to remove the right thyroid lobe. Pathological evaluation fit with follicular adenoma. Genetic testing of the resected tissue showed a somatic activating *TSHR* mutation that was not found in DNA derived from white blood cells. Postoperatively, the patient was treated with levothyroxine for 2 years because of transient central hypothyroidism due to suppression of the hypothalamus-pituitary-thyroid axis secondary to longstanding hyperfunction of the autonomous functioning thyroid nodules. Currently, thyroid function is normal without treatment.

This case illustrates the additional value of diagnostic imaging in cases of nonautoimmune thyrotoxicosis, especially when physical examination is suspicious for the presence of thyroid nodules. According to the latest ETA guideline, imaging is not necessary in the diagnostic workup of pediatric GD. However, when there are no signs of thyroid autoimmunity, thyroid ultrasonography and scintigraphy (preferably with ^99m^Tc-pertechnetate) are advised. To minimize radiation exposure in children, thyroid ultrasonography with Doppler blood flow assessment is preferred over scintigraphy (12). However, scintigraphy is better suited for diagnosing a “hot” autonomous nodule and excluding low iodine uptake thyrotoxicosis as illustrated by case 3 (3). Autonomous nodules caused by somatic mutations in *TSHR* are very rare in children. Somatic mutations activating *GNAS* or *EZH1* may cause autonomous thyroid hormone-producing nodules and are even more rare (13-15). Thyroid cancer is reported but is rare in autonomous functioning thyroid nodules in children (16). Activating germline mutations in *GNAS* may cause McCune–Albright syndrome resulting in a multinodular toxic goiter, where activating germline mutation in *TSHR* causes nonautoimmune hyperthyroidism with or without nodular lesions within the thyroid (3, 17, 18). Therefore, genetic testing of both the resected nodular tissue and DNA derived from white blood cells should be performed to distinguish between a somatic or germline *TSHR* mutation.

#### Case 4

A 15-year-old girl was evaluated by a pediatrician because of tiredness for several months. She had complaints of hair loss, heat intolerance, and palpitations during exercise and had lost 4 kg bodyweight in 2 months. Family history was negative for autoimmune thyroid disease. An initial biochemical workup showed a low serum TSH (0.01 mU/L; RI 0.5-5.0) and high FT4 (40 pmol/L; RI 12-22). Anti-TPO antibodies and TRAb levels were negative. The girl was referred to our center for further diagnostics and treatment of thyrotoxicosis. At presentation at our outpatient clinic, there was no tachycardia, and, because the complaints were relatively mild, treatment with ATD was not started yet. Biochemical reevaluation of thyroid hormone levels and TRAb levels showed a suppressed TSH (<0.01 mU/L), a lower FT4 level compared to initial presentation (26.5 pmol/L), a slightly elevated T3 (3.15 nmol/L; RI 1.3-2.7), and negative anti-TPO antibodies and TRAb levels. Measurement of TRAb levels was repeated as it is known that in cases of pediatric GD TRAb levels can be absent at initial presentation but become positive later on (3). Because FT4 levels lowered spontaneously, a wait-and-see policy was followed. Within 1 week, the FT4 level normalized, and the TSH was not suppressed anymore 6 weeks later. Thyroid function remained normal, and TRAb levels remained negative during follow-up. The probable diagnosis of mild subacute thyroiditis with a thyrotoxic phase was made.

Cases 3 and 4 illustrate that, however rare in pediatric patients, in “mild’ cases of thyrotoxicosis with minimal clinical and biochemical disturbance, a period of close monitoring can be very helpful to determine the need for treatment. Especially in pediatric patients with absent TRAb, this period of monitoring may give additional clinical and biochemical information leading to the establishment of the correct diagnosis. In case 3, the persistent mild thyrotoxicosis could be attributed to the presence of the “hot” nodules after additional diagnostic imaging, where in case 4 the spontaneous normalization of thyroid function pointed toward the diagnosis of mild subacute thyroiditis without any need for treatment. Subacute thyroiditis, also known as de Quervain thyroiditis, is an inflammatory condition of the thyroid presumably caused by a viral infection (19, 20). In the acute phase, biochemical evaluation may show high inflammatory markers (erythrocyte sedimentation rate and C-reactive protein) and thyroid hormone excess (3, 19). Subacute thyroiditis is a self-limiting condition that may recur. Patients with subacute thyroiditis may, but not always, go through a thyrotoxic phase that can be followed by a phase of transient hypothyroidism (19). In general, treatment with antithyroid drugs is not needed.

Pediatric thyrotoxicosis is a rare clinical condition, and even the most common cause, pediatric GD, is classified as a ORPHA.net rare disease. To optimize the clinical management and treatment outcome of pediatric GD patients, it has been recommended that a pediatric endocrinologist is involved in the care of all patients with pediatric GD (3). Cases 1 to 4 illustrate that pediatric endocrinologists with expertise in pediatric thyrotoxicosis have an important role in the diagnostic workup of challenging cases of thyrotoxicosis leading to an individually tailored diagnostic workup and may prevent incorrect diagnoses and unjustified start of ATD treatment. Table 2 provides an overview of the diagnostic findings and final diagnosis of the 4 presented cases.

**Table 2.** The biochemical profiles and final diagnoses of 4 of the cases of pediatric thyrotoxicosis

Case	Signs/symptoms	TSH (mU/L)	FT4 (pmol/L)	FT3	TRAb	Additional diagnostics	Diagnosis
**1**	Abdominal pain, no clear signs of thyrotoxicosis	1.16 (RI 0.5-5.0)	96.6 (RI 12-22)	N/A	Negative	Thyroid ultrasound: normal thyroid gland Genetic testing ruled out *THRB* mutation Normal pituitary MRI and low plasma alpha subunits ruled out TSH-producing pituitary adenoma Biochemical analysis thyroid hormones on other platform showed normal thyroid hormone levels (FT4 14.7; RI 10-12, TSH 1.8; RI 0.5-5)	Abnormal thyroid function tests due to assay interference
**2**	Weight loss	5.6 (RI 0.6-4.8)	48 (RI 12-22)	13 pmol/L (RI 3.0-6.2)	Negative	Genetic testing showed heterozygous pathogenic variant in *THRB*	Generalized thyroid hormone resistance
**3**	Tiredness, swelling in the neck, and tachycardia	<0.01 (RI 0.5-5)	24.6 (RI 12-22)	2.9 nmol/L (RI 1.3-2.7)	Negative	Negative for TPO and Tg antibodies Thyroid ultrasound: 2 nodules in the right lobe Scintigraphy: increased focal uptake at the location of the nodular lesions Genetic testing of resected thyroid tissue showed a somatic activating *TSHR* mutation	Autonomous functioning thyroid nodule due to somatic activating *TSHR* mutation
**4**	Tiredness, hair loss, heat intolerance, palpitations, and weight loss	0.01 (RI 0.5-5.0)	40 (RI 12-22)	3.15 nmol/L (RI 1.3-2.7)	Negative	Negative for TPO antibodies Repeatedly negative TRAb Normalization of FT4 and TSH levels without treatment during follow-up	Mild subacute thyroiditis with thyrotoxic phase

Abbreviations: FT3, free T3; FT4, free T4; MRI, magnetic resonance imaging; N/A, not applicable; RI, reference interval; Tg, thyroglobulin; TPO, thyroperoxidase; TRAb, TSH-receptor antibodies.

### Challenges in the Treatment of Pediatric GD

#### Case 5

**Figure 2.**
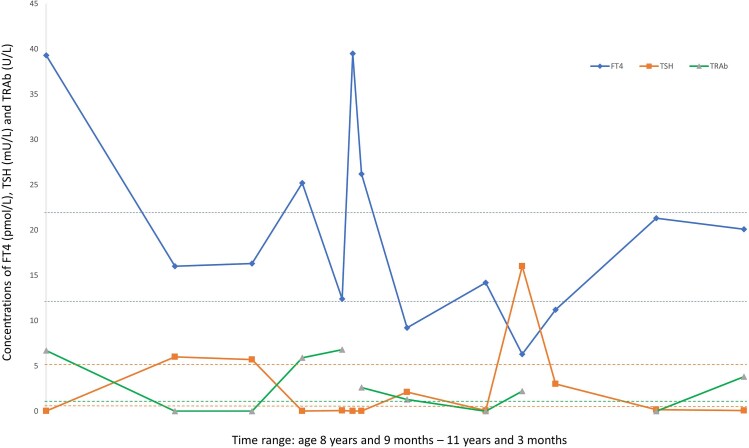
Graphical illustration of alterations in FT4, TSH, and TRAb concentrations over 30 months’ time in a girl with Graves’ disease with several immunological flareups during antithyroid drug treatment. Dotted lines illustrate the reference intervals for FT4, TSH, and TRAb. Assays did not change during the treatment period. Abbreviations: FT4, free T4; TRAb, TSH-receptor antibodies.

An 8-year-old girl was evaluated by a pediatrician because of suspected hyperthyroidism. She had a goiter, heat intolerance, and weight loss. The family history was positive for GD (father). Biochemical evaluation revealed overt hyperthyroidism [FT4 81.7 pmol/L; RI 12-22, free T3 (FT3) 15 pmol/L; RI 2.8-5.5, TSH <0.01 mU/L; RI 0.5-5]. Thyroid ultrasound showed an inhomogeneous enlarged thyroid gland. ATD treatment was started with methimazole. The girl was referred to our outpatient clinic for further evaluation and follow-up. Additional diagnostic testing revealed increased TRAb levels (6.7 U/L; RI 0-1.8), confirming the diagnosis of pediatric GD. Following the dose titration treatment strategy, the ATD dose was gradually lowered from 20 mg methimazole daily to 5 mg methimazole daily. Seven months after the start of ATD treatment TRAb levels were <1.0 U/L. Fifteen months after the start of ATD treatment, the girl was still on 5 mg methimazole daily, but she had an immunological flareup with increased TRAb levels (5.9 U/L) and recurrent biochemical hyperthyroidism (FT4 55.0 pmol/L; FT3 19 pmol/L; TSH <0.01 mU/L) requiring an increased ATD dose. After several months of treatment with higher doses of ATD, TRAb levels normalized and she became hypothyroid, and ATD dose could be lowered again. Thirty-four months after the initial start of ATD treatment, at the age of 11 years, she had a second immunological flareup and biochemical relapse of hyperthyroidism under dose-titrated ATD treatment. The ATD dose was increased, and the patient was counseled about definitive treatment options. Until now, the patient and her parents have decided to continue ATD treatment. The course of FT4, TSH, and TRAb concentrations over time in this case is illustrated in Fig. 2.

This case illustrates that an immunological flareup of GD may occur during ATD treatment. In the case of dose titration therapy, a reappearance or increase of TRAb levels may result in recurrent hyperthyroidism requiring intensified treatment. In contrast, an immunological flareup under block and replace therapy will not cause recurrent hyperthyroidism due to the complete suppression of thyroid hormone synthesis due to the higher ATD dose used. However, an important question is whether block and replace treatment only masks the presence of increased immunological activity associated with a lower permanent remission rate or whether treatment with higher methimazole doses in block and replace treatment actually has a beneficial effect on immunological remission. It has been previously suggested that methimazole itself has immunomodulatory and antioxidant effects (21, 22). Although this hypothesis may suggest that block and replace therapy may be beneficial for those patients who experience an immunological flareup under dose titration, the only randomized trial comparing block and replace and dose titration treatment strategies in pediatric GD did not report better biochemical stability compared to dose titration (23). Studies evaluating the remission rate in pediatric GD comparing block and replace vs dose titration are lacking. In this case, after immunological flareup it was decided to continue the dose titration treatment strategy to reduce the potential risk of ATD side effects that are associated with a higher ATD dose. Hypothetically it may have been better to choose to convert the treatment to a block and replace strategy to optimize the possible immunomodulatory effect of methimazole; however, evidence for this treatment strategy is scarce. An advantage of dose titration may be the identification of patients with recurrent immunological flareups under ATD treatment who may be less likely to achieve permanent immunological remission in the long term.

According to the current guideline, prolonged ATD treatment for a minimum of 3 years aims at reaching lifelong immunological remission and euthyroidism in pediatric GD. However, after 5 years of ATD treatment, a permanent remission rate of only 50% has been reported (24).

An important consideration to primarily aim for permanent functional and immunological remission in the treatment of pediatric GD is that this would result in lifelong euthyroidism without the need for medical treatment. In addition to the practical benefits of not having to use medication, there is evidence that long-term levothyroxine treatment, even in patients with normal TSH concentrations, is associated with impaired psychosocial well-being compared to controls (25). There are studies that report a reduced quality of life in patients who underwent RAI as definitive therapy (26). Nevertheless, even with a prolonged ATD treatment duration, still 50% or more of the pediatric GD patients require definitive treatment. Therefore there is an ongoing debate about whether patients would benefit from earlier definitive treatment (27, 28). In this perspective, it is interesting that in a study in young GD patients (< 30 years of age) who underwent definitive treatment, 36% reported that they would have preferred to have had definitive treatment earlier, as they believe they would have “felt normal sooner” (29). It is important to take into account that in young people diagnosed with and treated for GD during childhood, quality of life was found to be lower than that of healthy controls, particularly in the psychosocial domain (29). For some but probably not all of those patients, definitive treatment may result in improved quality of life (26, 30).

For now, it seems to be crucial to involve the patient and parents in shared decision making on the management of his/her condition, with taking individual disease characteristics and chances of achieving permanent remission into account.

#### Case 6

A 16-year-old boy was evaluated by a pediatrician because of suspected hyperthyroidism. His complaints included a tremor, heat intolerance, increased sweating, irritable behavior, and 5 kg weight loss. The diagnosis of GD was established after biochemical evaluation (FT4 > 100.0 pmol/L; RI 9-22, total T3 4.69 nmol/L; RI 1.17-2.52, TSH <0.01 mU/L; RI 0.27-4.20, TRAb 36.10 U/L; RI <0.55). ATD treatment was started using dose titration. In the first 2 months of ATD treatment, a gradual decrease of FT4 levels was observed (57.7-35.5 pmol/L). Biochemical evaluation 3 and 4 months after the start of ATD treatment showed a rise in FT4 level >100.0 pmol/L despite an increased methimazole dose of 60 mg/day (0.65 mg/kg/day). The boy was referred to our outpatient clinic for further evaluation. Suboptimal compliance was suspected as a cause for the rise in FT4 levels. He was initially treated with methimazole twice daily. To improve his compliance, the ATD frequency was changed to once daily, and he was asked to send self-made videos of ATD ingestion with his smartphone to the pediatrician via e-mail every day. Video-controlled ingestion of ATD with positive feedback by the treating pediatrician resulted in optimal compliance and consequently euthyroidism within 4 weeks.

This case illustrates that suboptimal compliance should always be considered an important reason of persistent hyperthyroidism despite high doses of ATD. Resistance to ATD is extremely rare and has only been reported in a few case reports (31). Patient compliance is especially challenging when dealing with teenagers and adolescents. In our outpatient pediatric endocrinology clinic, we have used daily self-made videos of drug ingestion successfully multiple times, both in the treatment of pediatric GD as well as in the treatment of persistent hypothyroidism in Hashimoto's disease. It has been reported previously that sending a video of every drug ingestion to obtain monetary rewards leads to better compliance in the treatment of hypertension (32). In our experience, frequent positive feedback on sending the videos via electronic messaging also has a positive effect on the patient's routine and results in improved compliance. In this era, where (almost) every patient has a smartphone to make self-made videos of drug ingestion, this is a cheap, and in our experience very effective, way of improving compliance in the treatment of pediatric GD. This prevents unnecessary additional diagnostic tests for potential drug malabsorption or ATD resistance and a potential hospitalization to clinically observe drug ingestion.

#### Case 7

A 15-year-old girl was diagnosed with hyperthyroidism due to GD. Treatment with ATD following the dose titration strategy was started with good results. She became euthyroid, but complaints of fatigue remained. During the first year of treatment, progressive arthralgia was reported. After 10 months of ATD treatment, it was discussed that both the arthralgia and persistent fatigue may be adverse effects of methimazole treatment. Total thyroidectomy as definitive treatment was considered as no alternative medical treatment of hyperthyroidism is available in children, due to contraindication for propylthiouracil in children. To be more certain that the complaints of fatigue and arthralgia could be attributed to ATD treatment, a trial off ATD was discussed. At that moment, the girl was euthyroid and TRAb levels normalized. Methimazole treatment was stopped, and the fatigue and arthralgia clearly improved. Unfortunately, after 3 weeks she showed recurrent hyperthyroidism and high TRAb levels. ATD treatment was restarted, and the complaints of fatigue and arthralgia returned. Subsequently, the girl underwent total thyroidectomy as definitive treatment. After surgery she had no complaints of fatigue or arthralgia and was euthyroid under levothyroxine substitution therapy.

Approximately 15% of pediatric patients diagnosed with GD and treated with ATD, mainly methimazole and carbimazole, experience at least 1 adverse effect or adverse event (24). Reported adverse effects of ATD treatment in pediatric patients include cutaneous reactions (incidence 11.2%), arthralgia/myalgia (1.4%), neutropenia/leukopenia (1.1%), liver transaminase elevation (1.0%), headache (0.8%), gastrointestinal complaints (0.5%), agranulocytosis (0.3%), hair loss (0.3%), fever (0.2%), and sore throat (0.2%) (24). The majority of adverse events occur within the first 3 months of treatment, with a higher incidence rate observed in younger children (33, 34). It is important to note that severe adverse events may be dose-dependent (35).

An increase in transaminase level (>3 times the upper limit of normal) and agranulocytosis are considered severe adverse events and should prompt the cessation of ATD and the start of counseling for definitive treatment strategies (5). Unfortunately, there is no alternative long-term medical treatment available for pediatric GD patients. Propylthiouracil is contraindicated in children due to the risk of irreversible hepatic failure (36). In cases of severe adverse events, patients should undergo definitive treatment (RAI or thyroidectomy). Thyroidectomy may be preferable over RAI since ATDs can be stopped immediately after thyroidectomy but need to be temporarily continued after RAI.

Mild adverse effects, such as rash and mild arthralgia, may resolve on their own. In such cases, symptomatic management with antihistamines or nonsteroidal anti-inflammatory drugs may be indicated. However, in case of severe arthralgia such as in the illustrated case, ATD needs to be stopped. A potential role in the development of arthralgia may be antineutrophil cytoplasmic antibody positivity that has been attributed to both GD itself as well as to its treatment with ATD (37, 38).

This case illustrates that complaints of malaise, like persistent fatigue and arthralgia, may be present in patients who are being treated with ATDs. These symptoms may be related to the ATD treatment itself (24). Before definitive treatment is considered, we suggest having a trial off ATD to evaluate the evolution of symptoms as illustrated by case 7. If symptoms disappear, this suggests that the complaints are related to ATD treatment. In that case, definitive treatment can be considered, especially in cases where a low likelihood of achieving permanent remission is suspected. Recurrent hyperthyroidism during the trial off ATD can be treated symptomatically using beta blockade if a longer trial off ATD is wanted.

Another challenging aspect in the management of patients with GD is the current differences between the pediatric and adult treatment guidelines (3, 12). While pediatric patients with newly diagnosed GD are medically treated for 3 years or longer, adult patients are treated with ATD for 12 to 18 months. If after this period TRAb levels are still high, ATD treatment is continued, with repeat TRAb measurement 12 months later. Alternatively, definitive therapy (RAI or thyroidectomy) is advised, according to the latest ETA guideline for the management of adult GD (12).

Solid evidence for the best medical treatment strategy in adolescence during the transition from pediatric to adult care is lacking. Looking back at our own experience in the last 10 years (2014-2023), we saw a total of 136 newly diagnosed pediatric GD patients at our tertiary pediatric endocrinology clinic; 22.8% of them (n = 31 out of 136) were 16 or 17 years of age at diagnosis and therefore encounter the aforementioned guideline differences. These adolescent patients should have an individually tailored treatment duration aiming at lifelong immunological remission. Definitive treatment before a minimum ATD treatment duration of 3 years can only be considered in cases with a very low likelihood of achieving immunological remission. Future treatment guidelines on (pediatric) GD should include evidence-based advice on how to treat young GD patients during transition from pediatric to adult care.

## Summary

The most common cause of pediatric thyrotoxicosis is GD. Nevertheless, it is important to consider other causes of pediatric thyrotoxicosis. Involvement of a pediatric endocrinologist may help in the diagnostic workup of these children since these other causes are very rare. For children diagnosed with GD, a recent clinical guideline is available. In this paper we discuss some challenges we experienced in the treatment of pediatric GD that are not captured by the guideline and may be helpful for clinicians facing challenging cases of pediatric GD.

## Data Availability

Data sharing is not applicable to this article as no data sets were generated or analyzed during the current study.
